# Comparison of spatio-temporal gait parameters between the GAITRite® platinum plus classic and the GAITRite® CIRFACE among older adults: a retrospective observational study

**DOI:** 10.1186/s12877-023-03811-7

**Published:** 2023-03-07

**Authors:** Guillaume Sacco, Grégory Ben-Sadoun, Jennifer Gautier, Romain Simon, Maude Goupil, Pauline Laureau, Jade Terrien, Cédric Annweiler

**Affiliations:** 1 Université Côte d’Azur, Centre Hospitalier Universitaire de Nice, Clinique Gériatrique de soins ambulatoires, Nice, France; 2 Université Côte d’Azur, CoBTek, Nice, France; 3 LPPL, Laboratoire de Psychologie des Pays de la Loire, Univ Angers, Université de Nantes, EA 4638 LPPL, SFR CONFLUENCES, Angers, F-49000 France; 4 Department of Geriatric Medicine and Memory Clinic, Research Center on Autonomy and Longevity, University Hospital, Angers, France; 5 Normandie Université, UNICAEN, INSERM, COMETE, CYCERON, CHU Caen, 14000 Caen, France; 6 School of Medicine, Health Faculty, University of Angers, Angers, France; 7 Robarts Research Institute, Department of Medical Biophysics, Schulich School of Medicine and Dentistry, The University of Western Ontario, London, ON Canada; 8 Centre de Recherche sur l’Autonomie et la Longévité (CeRAL), Service de Gériatrie, CHU d’Angers, 4, rue Larrey, 49933 Angers Cedex 9, France; 9 UMR-S 1075 Inserm, COMETE, Pôle des Formations et de Recherche en Santé, 2 Rue des Rochambelles, CS 14032, 14 032 CAEN Cedex, France

**Keywords:** *Instrumented walkway system*, *GAITRite*, *Older adults*, *Neurocognitive disorder*, *Falls*, *Walking aids*

## Abstract

**Background:**

The GAITRite® system is one of the gold standards for gait electronic analysis, especially for older adults. Previous GAITRite® systems were composed of an electronic roll-up walkway. Recently, a new GAITRite® electronic walkway, named CIRFACE, was commercialized. It is composed of a changeable association of stiff plates, unlike previous models. Are the gait parameters measured similar between these two walkways among older adults and according to the cognitive status, the history of falls, and the use of walking aids?

**Methods:**

In this retrospective observational study, 95 older ambulatory participants (mean, 82.6 ± 5.8 years) were included. Ten spatio-temporal gait parameters were measured simultaneously with the two GAITRite® systems in older adults while walking at comfortable self-selected pace. The GAITRite® Platinum Plus Classic (26’) was superimposed on the GAITRite® CIRFACE (VI). Comparisons between the parameters of the two walkways were performed using Bravais-Pearson correlation, between-method differences (corresponding to bias), percentage errors and Intraclass Correlation Coefficients (ICC_2,1_). Subgroup analyses were performed according to the cognitive status, the history of falls in the last 12 months and the use of walking aids.

**Results:**

The whole walk parameters recorded by the two walkways were extremely correlated with a Bravais-Pearson correlation coefficient ranging from 0.968 to 0.999, *P < .001*, indicating a very high correlation. According to the ICC_2,1_ calculated for absolute agreement, all gait parameters had excellent reliability (ranging from 0.938 to 0.999). Mean bias for 9 parameters out of 10 were ranged from − 0.27 to 0.54, with clinically acceptable percentage errors (1.2–10.1%). Step length showed a substantially higher bias (1.4 ± 1.2 cm), nevertheless the percentage errors remained clinically acceptable (5%).

**Conclusion:**

When walking at comfortable self-selected pace, the standard spatio-temporal walk parameters provided by both the GAITRite® PPC and the GAITRite® CIRFACE seem similar and very highly correlated in older adults with various cognitive or motor status. The data of studies using these systems can be compared and mixed with a very low risk of bias in a meta-analytic process. Also, the geriatric care units can choose the most ergonomic system according to their infrastructure without affecting their gait data.

**Trial registration:**

NCT04557592 (21/09/2020).

**Supplementary Information:**

The online version contains supplementary material available at 10.1186/s12877-023-03811-7.

## Introduction

The GAITRite® system is an instrumented gait analyzer with resistive pressure sensors. It was used to analyze gait parameters in younger, older and geriatric populations [1, 2] well as in patients suffering from minor or major NeuroCognitive Disorders (NCD) related to various etiologies such as Alzheimer Disease (AD), vascular or mixed diseases [3, 4], but also Parkinson disease [5, 6]. During the two past decades, a growing number of studies investigated relationships between spatio­temporal gait parameters and age, gender [7–9], domain-specific cognitive decline (e.g. declines of episodic memory and executive functions) [10], frailty [11, 12], history of falls [4, 13], use of walking aids including canes [13, 14] and rollators [15], and lower-limb characteristics like physical capacities [15, 16] or lower-limb length [17]. These researches reinforce the interest of providing a robust gait analysis of the older patients during standard evaluation in medical practice. Walking 4 to 14 m at comfortable self-selected pace was the condition most frequently studied so far. Other conditions can be investigated such as walking as fast and safe as possible, walking as slow as possible or walking at its own speed while performing a cognitive task simultaneously (i.e., dual-task paradigm).

To our knowledge, first studies on accuracy and reliability of GAITRite® were performed in 2001 in a case study among younger women [18]. Since 2005, the GAITRite® was considered as one of the gold standards in gait analyses with very high accuracy and reliability in older adults [19]. This technology was also considered as gold standard for metrological validation studies of other sensors like the ones wearable (laser [20], pressure [21]), ambient (ultrasound [22]) or vision based (depth camera, smartphone camera [23], infrared camera and webcam [24]). During the two past decades, the GAITRite® system was composed of a roll-up electronic walkway (e.g., Platinum Plus Classic (PPC), RE, Basic and Safari) with a length ranging from 4 to 10 m, 0.9 m wide and 0.5 cm high. The walkway encapsulated multiple resistive pressure sensors (between 14,000 and 30,000 sensors depending on the walking length) arranged in a grid with a spatial resolution of 1.27 cm and a sampling frequency from 60 to 240 Hz (see for example: https://biometrics.fr/web/24-gaitrite-et-cirface).

Since 2016, GAITRite® has commercialized a new system named CIRFACE. This new system is based on the same technology as the roll-up walkway (multiple resistive pressure sensors arranged in a grid with a spatial resolution of 1.27 cm) but the walkway is composed of a changeable association of stiff plates. This technology has the ergonomic advantage of being more adaptable to variable spatial organizations from one geriatric care unit to another compared to the roll-up walkway GAITRite® (which is not adjustable in length). Note that beyond the analysis of walking on a straight line, this system can analyze several walking situations, including turning around, with and without obstacles, going up and down stairs and other more complex walks such as ZigZag, T, L, 8 and circle. Until now, only two studies have used this recent walkway system, the first one in patients with incomplete spinal cord injury [25], and the second one in patients with multiple sclerosis [22].

To ensure comparability between the results of studies using these different walkways (e.g., in a meta-analytic process), to confirm that their respective external structure does not influence the result of the gait analysis and, to confirm the lack of impact between using a roll-up walkway GAITRite® or the GAITRite® CIRFACE among older adults, it may be relevant to compare directly the performance of both walkways. In the geriatric population (including inpatients), cognitive decline is a very common pathology [26] and it influences walk parameters. Oliveira et al. [27] showed differences in velocity, gait cycle time, step length, and cadence during a walk at self-selected pace between Cognitively Healthy Individuals (CHI) and patients with mild cognitive impairment (i.e., minor NCD), compared to patients with AD. Velocities are often comprised between 0.95 and 1.25 m.s^− 1^ for CHI and patients with minor NCD [1, 2, 8, 9, 17, 27, 28], whereas they are lower for major NCD (often under 0.95 m.s^− 1^ [27–29]). In addition, the results from Savica et al. [30] suggested that the decline in spatiotemporal gait parameters during a walk at self-selected pace (e.g., velocity, cadence, stride length, swing time, stance time, support base) were all predictive of the decline in memory, executive, language and visuospatial abilities in older patients with various neural and vascular disorders. In the same way, falls are also a very common condition in geriatric patients with potential severe consequences on gait parameters [4, 13]. Walking with a walking aid changes gait parameters [31] and capturing gait parameters with a walking aid should be a technological issue that should also be challenged [13, 14]. Older patients with history of fall and/or using a walking aid have low velocities (under 0.85 m.s^− 1^ [13, 14]). Last, these cognitive and motor declines may be associated. Patients with a major NCD and fall history have the most severe gait impairments (e.g., velocities under 0.65 m.s^− 1^ [13, 29]).

Taken together, it may be relevant to compare the roll-up walkway GAITRite® and the GAITRite® CIRFACE in a sample of older (in)patients, including several severities of cognitive and motor declines.

Thus, the main objective of the present study was to compare the standard spatio-temporal gait parameters measured with a roll-up walkway GAITRite® and the GAITRite® CIRFACE among older inpatients. Due to the heterogeneity of inpatients in geriatric care unit, and the great interest of provide a robust gait evaluation in this type of medical unit, the secondary objectives were to compare gait parameters according to: (i) the cognitive status, (ii) the history of falls, and (iii) the use of a walking aid.

## Methods

### Study design

The design of this study was observational and retrospective. It was conducted in the geriatric day care and memory consultation units of a French University Hospital between June 17, 2019 and February 26, 2020. In these two units, patient’s walk was systematically assessed using the GAITRite® as usual assessment to complete the geriatric and cognitive assessments.

### Participants

The population of the study was composed of older ambulatory participants aged over 74 and with various NCD status (without NCD, with minor NCD, with major NCD due to AD), with or without history of falls in the last 12 months, and with or without walking aid. Data were extracted if the participant did not refuse the use of its data.

### Apparatus

The GAITRite® PPC (26’) roll-up electronic walkway was used in this study (CIR Systems Inc., USA). Its dimensions were 884 cm long (793 cm active length), 90 cm wide (61 cm active width) and 0.6 cm high. It was compared to the GAITRite® CIRFACE (VI) electronic walkway composed of 12 plates (CIR Systems Inc., USA). Its dimensions were 576 cm active length (48 cm per plate), 111 cm wide (96 cm active width), 5.5 m² in total active area, and 3 cm height.

### Procedure

**Fig. 1:**
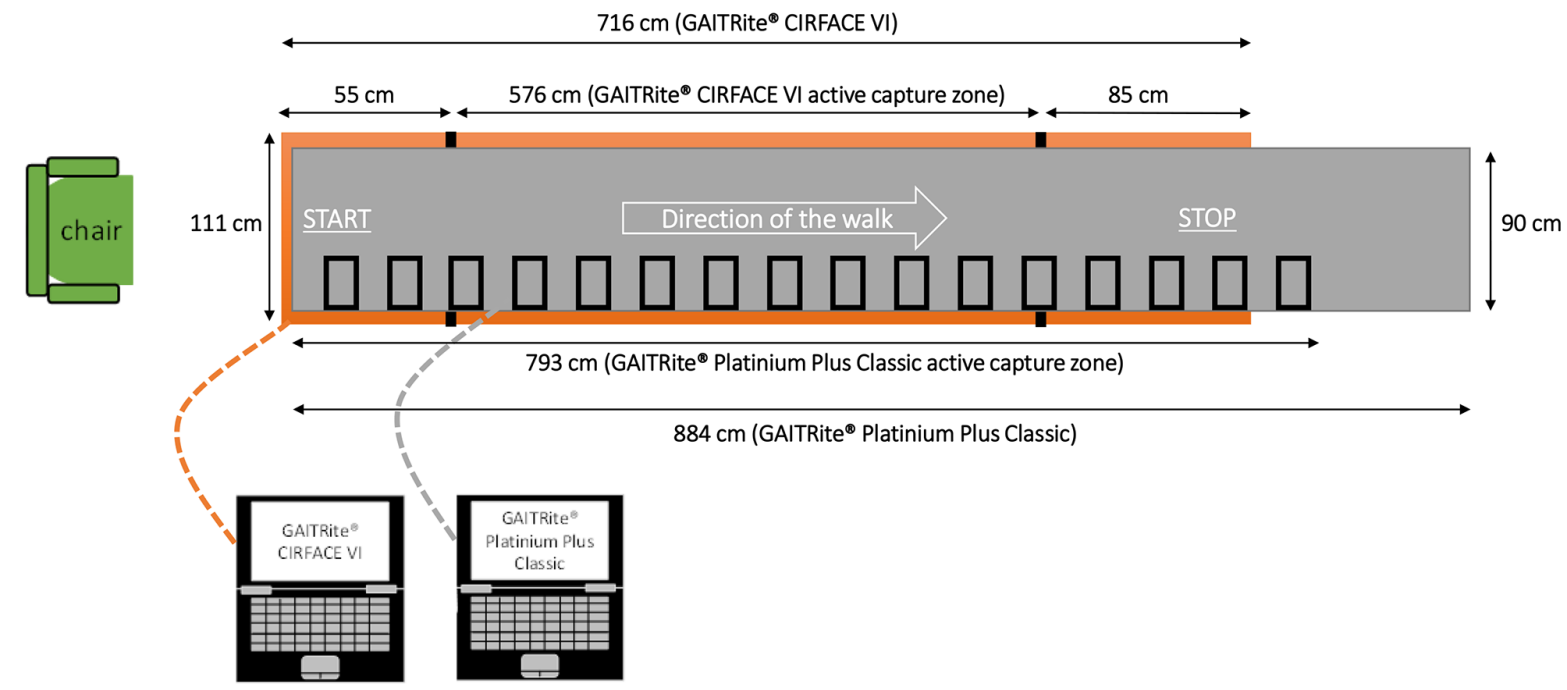
Experimental design (overview). The GAITRite® CIRFACE is in orange color. The GAITRite® Platinium Plus Classic is in grey

The two versions of the GAITRite® walkways were superimposed (see Fig. 1 to view experimental design). The GAITRite® CIRFACE was placed directly on the ground. The active capture zone of walk was 576 cm long. In order to secure the participant and to limit the effects of acceleration and deceleration at the beginning and at the end of the walk, an additional plate of the GAITRite® CIRFACE, without sensor, was placed before (55 cm long) and after (85 cm long) the GAITRite® CIRFACE active capture zone. The GAITRite® PPC was placed on the CIRFACE, starting at the same level as the GAITRite® CIRFACE’s non-active capture zone, and finishing at 253 cm beyond the active capture zone. Both GAITRite® systems were initialized before the patient started to walk. Thereby, patient’s walk was recorded by both walkways at the same time. Before starting to walk, the patient was sited in front of the walkway to prevent pre-fatigue. Then, the professional caregiver (nurse and/or medical student) asked him to stand up and to stand on the first plate without sensor. Then, he instructed him to walk on the walkway at his usual, comfortable speed (including cane or rollator if necessary) to the mark (which corresponded to the last plate without sensor of the GAITRite® CIRFACE). One trial was recorded of each patient.

### Data collected

#### Clinical measurements

Participants’ characteristics were collected during the consultation by a medical doctor and/or a neuropsychologist, including age, gender, weight, the use of a walking aid, history of falls in the last12 months, Mini Mental State Examination score (MMSE, scored over 30, the higher the better, cut-off 26) [32], Frontal Assessment Battery score (FAB, scored over 18, the higher the better, cut-off 15) [33], and mini Geriatric Depression Scale (mini GDS, scored over 4, the lower the better, cut-off 1) [34]. Note that to establish a precise cognitive diagnosis, the medical doctor and/or the neuropsychologist performed, as usual assessments, the MMSE, the FAB, the mini GDS, but also the basic and the instrumental activities of daily living. Depending of the results of these first explorations, they could complete the assessments using other cognitive tests more specific to a cognitive function (e.g., fluency tests, memory tests, cognitive flexibility tests) or to behaviors (e.g., delirium, apathy, mood disorders). With this strategy, they could establish a diagnosis of probable NCD and/or AD.

NCD status was collected to differentiate three subgroups: Cognitive Healthy Individual [CHI], minor NCD and AD.

#### Gait measurements

Spatio-temporal gait parameters were collected from the two GAITRite® systems, including the following standard parameters: velocity, cadence, step time, step length, stride time, stride length, support base, swing time, stance time and stride velocity. These gait parameters were chosen because of their relevance in characterizing the gait of older adults in two dimensions (length and width) [4, 8, 35].

Each GAITRite® system was connected to its own computer but they used the same gait analysis software produced by GAITRite®. Footprints were automatically detected and recorded by the software. Then, to check the correspondence of the footprint acquisitions between the two systems, the walks from the GAITRite® PPC were manually compared to the walks from the GAITRite® CIRFACE, and manually post-treated to have comparable data. For example, the first(s) and last(s) step(s) from the PPC were manually deleted to start the record with the same foot and to match the number of steps of the CIRFACE.

All the data collected were centralized by a data manager.

### Statistical analyses

In order to maximize the chance to obtain a normal distribution of the data to use parametric tests, and to compare three subgroups of patients according to their cognitive status, it was necessary to include 30 participants per group, i.e., 90 participants in total. The sampling method was probability sampling in which the sample was stratified according to the cognitive status of the patients (3 stratifications). A 10% margin of error was added to take in account possible technical concerns. Thus, we had to include 100 participants.

Categorical variables were described using numbers and percentages, and quantitative variables using means and standard deviations, as appropriate. Comparisons among the different cognitive status, among participants with and without history of fall and among participants with and without walking aids were performed using Chi² test for categorical variables (or exact Fisher test if appropriate), and Kruskal-Wallis analyze of variance for independent sample or Student *t* test for quantitative variables (or Mann-Whitney U test if appropriate). Correlations between the parameters of the two walkways were performed using Bravais-Pearson correlation. Between-method differences (± Standard Deviation [SD]), corresponding to bias, were presented with 95% Limits Of Agreement (LOA) calculated as:

1 $$LOA = mea{n_{bias}} \pm \,1.96 \times S{D_{bias}}$$

using the Bland-Altman method [36].

Percentage errors (PE) were calculated as:

2 $$\begin{gathered} pe = 100 \times (1.96 \times S{D_{bias}})/ \hfill \\((mea{n_{GAITRite{\kern 1pt} PPC}} + mea{n_{GAITRite{\kern 1pt} {\kern 1pt} CIRFACE}})/2) \hfill \\ \end{gathered} $$

and were considered clinically acceptable if < 30% [37].

Intraclass correlation coefficients (ICC_2,1_) were calculated to determine the absolute agreement between the mean gait parameters measured by the two systems. To interpret ICC values we used benchmarks suggested by Shrout and Fleiss [38] (> 0.75 excellent reliability, 0.40–0.75 fair-to-good reliability and < 0.40 poor reliability). A two -sided P-value < 0.05 was considered significant. Every statistical analysis was performed by a scientist and with SPSS (version 20; SPSS, Inc., Chicago, IL).

## Results

### Participants

One hundred and two participants were preselected. Four of them (1 CHI and 3 patients with minor NCD) were excluded because of missing data and three of them because of walk data which appeared miss-recorded. Finally, the study included 95 patients which represent 190 walks to compare. The mean age was 82.6 ± 5.8 years old, and 67 patients (70.5%) were women. The mean weight was 65.6 ± 14.0 kg, and the mean velocity (i.e., the walking speed) was 82 ± 22 cm/s. Among the participants, 14 (14.7%) walked with a walking aid and 59 (62.1%) fell at least once in the last 12 months. Regarding their cognitive functions, the mean MMSE was 23.4 ± 4.9, the mean FAB was 14.3 ± 3.0 and the mean mini GDS was 0.4 ± 0.7. Statistical comparisons between the participants according to their cognitive, fall and walking aid status were detailed in Table 1. CHI sub-group had 12.1% of participants using a walking aid and 78.8% of participants with a history of fall. Minor NCD sub-group had 11.5% of participants using a walking aid and 34.6% of participants with a history of fall. AD sub-group had 19.4% of participants using a walking aid and 66.7% of participant with a history of fall (see Additional File 1).

**Table 1:** Description of the participants included in the study

	Whole sample (N = 95)	Groups according to cognitive status				Groups according to falls status			Groups according to walking aid status		
		CHI (n = 33)	Minor NCD (n = 26)	AD (n = 36)	*P-value* ^‡^	No falls (n = 36)	Falls (n = 59)	*P-value* ^$^	No aid (n = 81)	Walking aid (n = 14)	*P-value* ^$^
Age (years)	82.6 ± 5.8	80.9 ± 6.3	82.6 ± 5.2	84.1 ± 5.5	0.064	81.1 ± 6.2	83.5 ± 5.5	0.056	81.9 ± 5.6	86.8 ± 5.4	0.003
Female gender	67 (70.5)	21 (63.6)	17 (58.6)	31 (86.1)	0.030	23 (63.9)	44 (74.6)	0.268	55 (67.9)	12 (85.7)	0.220
Weight (kg)	65.6 ± 14.0	70.8 ± 15.1	65.6 ± 12.6	60.9 ± 12.4	0.029	67.2 ± 13.2	64.7 ± 14.4	0.380	65.4 ± 13.6	66.9 ± 16.5	0.785
Velocity (cm/s)	81.6 ± 21.8	90.0 ± 24.1	76.5 ± 20.6	77.6 ± 18.4	0.033	83.0 ± 21.8	80.8 ± 22.0	0.631	85.2 ± 21.1	60.9 ± 12.9	< 0.001
Walking aid	14 (14.7)	4 (12.1)	6 (20.7)	7 (19.4)	0.622	2 (5.6)	12 (20.3)	0.049	0 (0)	14 (100)	-
Fall at least one time in the last 12 months	59 (62.1)	26 (78.8)	12 (41.4)	24 (66.7)	0.002	0(0)	59(100)	-	47 (58.0)	12 (85.7)	0.049
MMSE (/30)*	23.4 ± 4.9	27.1 ± 2.4	24.9 ± 2.9	19.2 ± 4.6	< 0.001	23.3 ± 4.8	23.4 ± 5.1	0.931	23.7 ± 4.8	20.9 ± 5.3	0.070
FAB (/18)†	14.3 ± 3.0	16.0 ± 2.8	14.6 ± 2.5	12.7 ± 2.9	< 0.001	14.4 ± 2.8	14.2 ± 3.2	0.720	14.5 ± 2.9	12.6 ± 3.4	0.085
Mini GDS (/4)	0.4 ± 0.7	0.3 ± 0.6	0.6 ± 0.8	0.3 ± 0.7	0.033	0.3 ± 0.6	0.4 ± 0.8	0.905	0.4 ± 0.7	0.4 ± 0.6	0.870

Data are presented with mean ± standard deviation (m ± sd) or number and percentage n(%) as appropriate; AD: Alzheimer Disease; CHI: Cognitive Healthy Individual; FAB: Frontal Assessment Battery; Mini GDS: Mini Geriatric Depression Scale; MMSE: Mini Mental State Examination; NCD: NeuroCognitive Disorder

*: 6 data were missing

†: 20 data were missing

‖: data from the CIRFACE walkway were presented are presented in cm/s

^‡^ : Comparison were performed using Chi square test or Fisher exact test for categorical variables, Kruskal-Wallis analyze of variance for quantitative variables

^$^ : Comparison were performed using Chi square test or Fisher exact test for categorical variables, Student T test or Mann-Whitney test for quantitative variables

### Gait measurements on the whole sample

Mean biases for 9 parameters out of 10 including velocity, cadence, step time, stride time, stride length, support base, swing time, stance time and stride velocity, were ranged from − 0.27 to 0.54, with clinically acceptable percentage of errors (1.2–10.1%, Table 2). Step length showed a substantially higher bias (1.37 ± 1.19 cm), nevertheless the percentage of errors remained clinically acceptable (4.7%, Table 2). There were no systematic differences in favor of an overestimation or underestimation of one system compared to the other (Table 2).

**Table 2:** Comparison between the roll-up and the plate walkways

	Roll-up system GAITRite® PPC Mean (SD)	Plates system GAITRite® CIRFACE Mean (SD)	Bias* Mean (SD)	95% LOA	PE	Pearson Corr.	ICC_(2,1)_
Velocity (cm/s)	81.07 ± 21.48	81.61 ± 21.81	0.54 ± 2.01	-3.41; 4.49	4.9	0.996	0.995
Cadence (step/min)	97.77 ± 12.87	97.97 ± 12.99	0.20 ± 1.08	-1.91; 2.32	2.2	0.997	0.996
Step time (s)	0.63 ± 0.09	0.62 ± 0.09	0.00 ± 0.01	-0.01; 0.01	1.9	0.998	0.998
Step length (cm)	49.42 ± 9.69	50.79 ± 9.16	1.37 ± 1.19	-0.96; 3.70	4.7	0.994	0.982
Stride time (s)	1.25 ± 0.13	1.24 ± 0.19	0.00 ± 0.01	-0.02; 0.02	1.6	0.999	0.998
Stride length (cm)	99.32 ± 19.39	99.04 ± 19.37	-0.27 ± 0.61	-1.48; 0.93	1.2	0.999	0.999
Support base (cm)	10.72 ± 3.80	10.76 ± 3.70	0.04 ± 0.55	-1.04; 1.13	10.1	0.989	0.989
Swing time (s)	0.42 ± 0.05	0.41 ± 0.05	-0.01 ± 0.01	-0.04; 0.01	6.0	0.968	0.938
Stance time (s)	0.82 ± 0.15	0.83 ± 0.15	0.01 ± 0.01	-0.02; 0.04	3.1	0.996	0.994
Stride velocity (cm/s)	82.01 ± 21.84	81.96 ± 22.14	-0.05 ± 1.41	-2.80; 2.70	3.4	0.998	0.998

* Difference between Roll-up and Plates system (Plates-Roll-up)

LOA : Limits Of Agreement

PE : Percentage Error

cm: centimeter; m: meter; min: minute; s: second

ICC : Intraclass Correlation Coefficient for absolute agreement

All Pearson correlations and ICCs were significant at P < .001

**Fig. 2:**
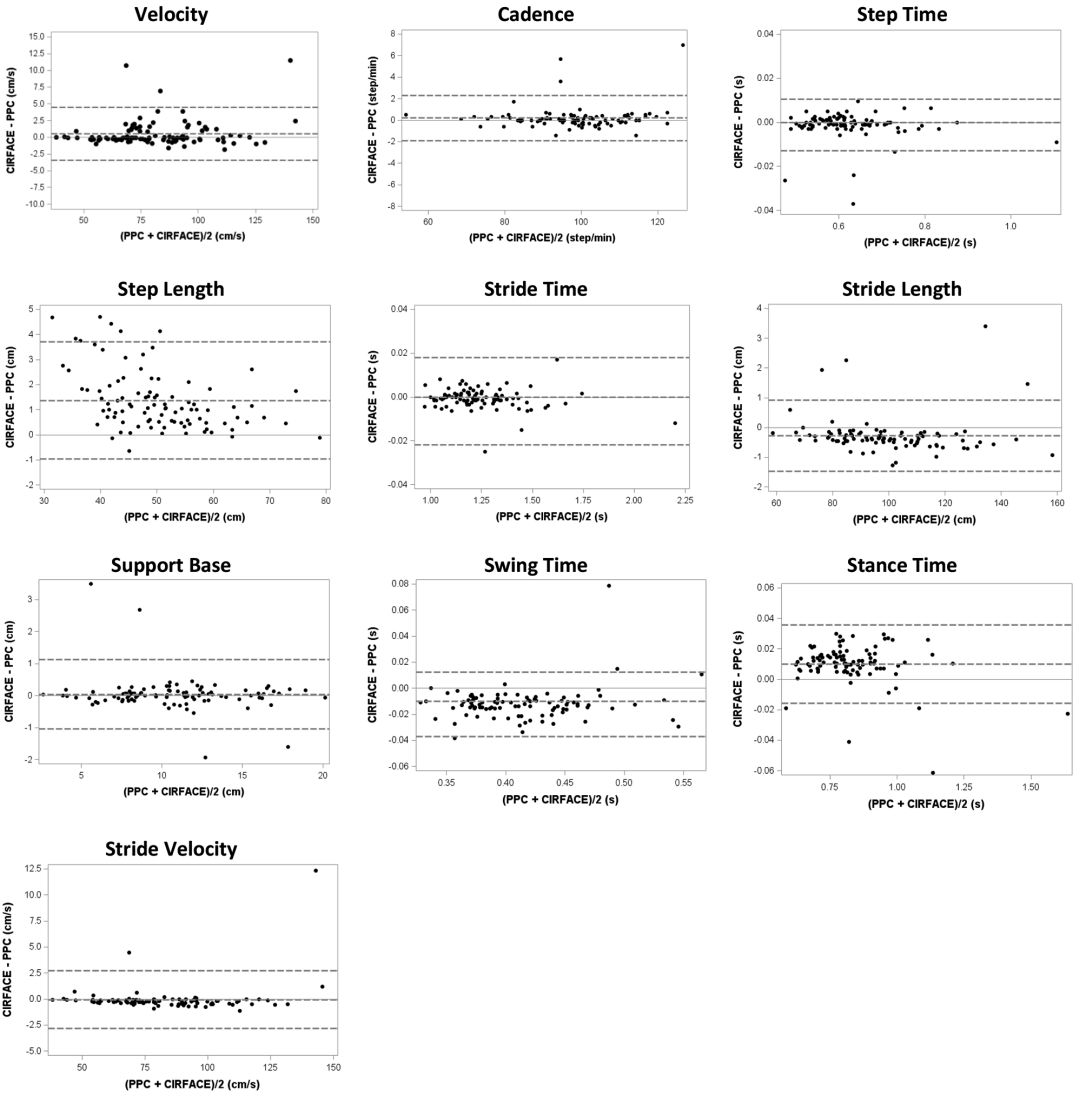
Bland-Altman plots for comparison between the Roll-up system GAITRite® PPC and the Plates system GAITRite® CIRFACE. Dotted lines indicate bias (mean difference between the 2 methods) and also upper and lower 95% limits of agreement (± 1.96 SD of the bias)

Limits of agreement were considered narrow for the whole set of gait parameters (Fig. 2).

All of the walk parameters recorded by the two walkways were extremely correlated regarding velocity, cadence, step time, step length, stride time, stride length, support base, swing time, stance time, and stride velocity with a Bravais-Pearson correlation coefficient ranging from 0.968 to 0.999, *P < .001*, indicating a very high correlation (Table 2). According to the ICC_2,1_ calculated for absolute agreement, all gait parameters had excellent reliability (ranging from 0.938 to 0.999, Table 2).

The GAITRite® CIRFACE provides similar data to the GAITRite® PPC in the whole sample.

### Gait measurements according to cognitive status

Mean biases, LOA and PE were very similar to those of the whole sample (see Additional File 1). All of the walk parameters according to cognitive status were very highly correlated for CHI, participants with Minor NCD and participants with AD (respectively, Bravais-Pearson correlation coefficient ranging from 0.976 to 0.999, *P < .001*, from 0.943 to 1, *P < .001 and* from 0.980 to 1, *P < .001* ; ICC_2,1_ ranging from 0.946 to 0.999, from 0.929 to 1 and 0.944 to 0.999, Table 3).

**Table 3:** Correlations between the roll-up and the plate walkways according to cognitive status

	CHI		Minor NCD		AD	
	Pearson Corr.	ICC_(2,1)_	Pearson Corr.	ICC_(2,1)_	Pearson Corr.	ICC_(2,1)_
Velocity	0.994	0.994	0.999	0.999	0.994	0.994
Cadence	0.995	0.995	1	1	0.995	0.995
Step time	0.999	0.999	1	1	0.995	0.995
Step length	0.996	0.989	0.991	0.967	0.992	0.977
Stride time	0.999	0.999	1	1	0.997	0.997
Stride length	0.999	0.999	1	0.999	1	0.999
Support base	0.976	0.974	0.996	0.997	0.995	0.995
Swing time	0.985	0.946	0.943	0.929	0.980	0.944
Stance time	0.998	0.996	0.995	0.993	0.994	0.991
Stride velocity	0.997	0.996	1	1	0.999	0.999

AD: Alzheimer Disease; CHI: Cognitive Healthy Individual; NCD: NeuroCognitive Disorder

All Pearson correlations and ICCs were significant at P < .001

The GAITRite® CIRFACE provides similar data to the GAITRite® PPC regardless of the cognitive status of the participants.

### Gait measurements according to walking aid

Mean biases, LOA and PE were very similar to those of the whole sample (see Additional File 1). All of the walk parameters according to walking aid status were very highly correlated for participants without walking aid and those using walking aid (respectively, Bravais-Pearson correlation coefficient ranging from 0.963 to 0.999, *P < .001 and* from 0.973 to 1, *P < .001*; ICC_2,1_ ranging from 0.930 to 0.999 and from 0.869 to 1, Table 4).

**Table 4:** Correlation between the rollup and the plate walkways according to walking aid status

	No aid		Walking aid	
	Pearson Corr.	ICC_(2,1)_	Pearson Corr.	ICC_(2,1)_
Velocity	0.995	0.994	1	1
Cadence	0.996	0.996	1	0.999
Step time	0.997	0.997	1	1
Step length	0.994	0.983	0.973	0.869
Stride time	0.998	0.998	1	1
Stride length	0.999	0.999	1	0.999
Support base	0.989	0.988	0.993	0.993
Swing time	0.963	0.930	0.990	0.975
Stance time	0.995	0.991	1	0.998
Stride velocity	0.998	0.998	1	1

All Pearson correlations and ICCs were significant at P < .001

The GAITRite® CIRFACE provides similar data to the GAITRite® PPC regardless of the walking aid status of the participants.

### Gait measurements according to fall status

Mean biases, LOA and PE were very similar to those of the whole sample (see Additional File 1). All of the walk parameters according to fall status were very highly correlated for participants with at least one fall for the last 12 months and participants without fall for the last 12 months (respectively, Bravais­Pearson correlation coefficient ranging from 0.989 to 0.999, *P < .001 and* from 0.957 to 1, *P < .001*; ICC_2,1_ ranging from 0.950 to 0.999 and from 0.927 to 0.999, Table 5).

**Table 5:** Correlation between the roll-up and the plate walkways according to falls status

	Non faller		Faller	
	Pearson Corr.	ICC_(2,1)_	Pearson Corr.	ICC_(2,1)_
Velocity	0.995	0.995	0.997	0.996
Cadence	0.996	0.996	0.997	0.997
Step time	0.997	0.997	0.999	0.999
Step length	0.992	0.979	0.994	0.983
Stride time	0.998	0.998	0.999	0.999
Stride length	0.999	0.999	1	0.999
Support base	0.989	0.989	0.990	0.990
Swing time	0.989	0.950	0.957	0.927
Stance time	0.996	0.993	0.997	0.995
Stride velocity	0.999	0.999	0.997	0.997

All Pearson correlations and ICCs were significant at P < .001

The GAITRite® CIRFACE provides similar data to the GAITRite® PPC regardless of the fall status of the participants.

## Discussion

### Comparison between Systems

This validation study showed that the GAITRite® CIRFACE provides similar data to the GAITRite® PPC for the spatio-temporal gait parameters of velocity, cadence, step time, step length, stride time, stride length, support base, swing time, stance time and stride velocity. Mean biases and PE were extremely small to consider differences between systems. Moreover, these differences were not systematically in favor of an overestimation or underestimation of one system compared to the other. It seems difficult to suggest an effect of the superposition of the two systems. These differences seem to be due to a random effect.

This study demonstrates that all of these spatio-temporal gait parameters provided both by the GAITRite® PPC and the GAITRite® CIRFACE were very highly correlated (with r ≥ .943 and ICC_2,1_ ≥ 0.927, *P < .001*) whether in the total population or in subgroups (CHI, participants with Minor NCD or AD, participants using a walking aid or not, and participants with history of falls or not). These results were higher, as expected, to those comparing GAITRite® systems to other gait parameter analysis systems [20–24], which used GAITRite® systems as reference tool (PPS and CIRFACE). The main explanation is that both GAITRite® PPC and CIRFACE systems use the same technology, and this tend to confirm that their respective external structure does not influence the result of the gait analysis. Note that vision-based sensors appear to be another type of technology capturing spatiotemporal gait parameters with relevance [23, 24], but GAITRite® systems appears to be the technology that can compute the most gait parameters.

### Gait parameters in our studied populations

Gait parameters in our study seem to describe poor performances in all our subgroups comparing to literature studies. To clarify our discussion, we will give examples only on velocity parameter, which substantially reflected gait performances. Indeed, this gait parameter seems to be the most relevant to distinguish older persons according to their cognitive status, fall history, and walking aid use [4, 13, 27]. To our knowledge, only Roman de Mettelinge & Cambier [13] reported lower velocities in patients living in residential aged care facilities with similar age and cognitive level than those of our study (velocities without walking aid equal to 67 cm/s and velocities with walking aid equal to 42.5 cm/s).

Concerning our CHI, velocities were 90 cm/s. Most studies reported higher velocities for CHI of similar age (between 100 and 120 cm/s) [1, 2, 8, 9]. Two hypotheses could underlie these results. Firstly, our experimental design did not provide sufficient precautions to exclude, on gait parameters analysis, the effects of acceleration and deceleration phases during walking due to the initiation and stop of the walk. Indeed, studies on CHI cited above ordered patients to start and stop walking at a distance comprised between 1 and 2 m of active capture zone, comparing to 55 and 85 cm in our study. As a result, in the conditions of our study, some recorded gait data (such as average the velocity) during the walk at comfortable self-selected pace over a distance of approximately 6 m may have been artificially underestimated. It would have been interesting to increase the walking distances before and after the active capture zone to better compare our results with other studies. Secondly, our study was conducted in geriatric units of a university hospital, mainly taking care of older adults with physical declines. Indeed, 78.8% of CHI had a history of fall, which is particularly high. Moreover, in addition to CHI with history of falls or those using a walking aid, some of CHI could present physical frailty syndrome undiagnosed. Other studies including patients with physical frailty syndrome reported similar velocities but all patients presented a cognitive decline [11, 12]. It would have been interesting to test the physical capacities of the lower limbs and/or to diagnose the presence of frailty syndrome in our study to confirm this second hypothesis.

Concerning our patients with minor NCD, velocities were 76.5 cm/s. Our results seem to be close to those reported in the literature, within the limit of finding comparable age groups. The studies including septuagenarian patients with MCI (between 73 and 76 years old) [17, 28, 39], reported higher velocities (between 95 and 117 cm/s) whereas those including octogenarian patients with MCI reported similar velocities. For example, Goyal et al. [40] reported a velocity of 80 cm/s in their 78-years-old patients with MCI, and De Cock et al. [17] reported a velocity of 76 cm/s in their over-80-years-old patients with MCI. Due to greater precautions also taken in these studies design compared to our study (the start and stop of the walk between 1 and 2 m of active capture zone), we can hypothesize that the inclusion of acceleration and deceleration phases in the gait analysis have less impact when gait performances are poorer.

Concerning our patients with AD, velocities were 77.6 cm/s. Our results seem like those reported in the literature, within the limit of finding comparable groups of disease severity. Callisaya et al. [28] reported similar velocities in patients with mild AD and MMSE score at 22/30. Finally, Callisaya et al. [28] and Camicioli & Licis [29] reported lower velocities in patients with moderate AD and MMSE score at 15–16/30.

### Limits of the study

From a metrological point of view in human movement analysis and considering the discussions above, this study presents some methodological limitations. However, it is important to note that this study aimed to compare two GAITRite® systems which used the same technology. The study interest was to know if standard gait parameters were similar between these two systems to allow future inter-study comparisons. Also, the choice of a heterogeneous geriatric population allowed us to verify these similarities in a sample with a wide range of cognitive and motor status. this heterogeneity is a major characteristic of geriatric care units. Our experimental design seems sufficient to answer the present research question, that is reflected by the low mean bias and the very high correlations found (r ≥ .943 and ICC_2,1_ ≥ 0.869) for all spatio-temporal gait parameters.

However, even if this study didn’t analyze directly the validity of the GAITRite® CIRFACE system, but rather analyzed this level of similarity with an electronic roll-up walkway GAITRite® (already accepted as gold standard), to extend the comparison at his extreme robustness, it would have been interesting to include in our study several elements used to validate this type of technology, especially: (i) more walking conditions (fast and slow walking conditions) to confirm the correlation levels in every circumstances; (ii) more distance to cover when walking per trial which would include more steps in the analysis (8 to 10 m); (iii) more precautions to exclude acceleration and deceleration phases of active capture zone (the start and stop of the walk 2 m before and after); (iv) more trials for each walking condition (3 trials) to test repeatability; and (v) more physical lower limb examinations (frailty diagnostic tests) to facilitate results interpretation between our subgroups and literature results. Yet, such an experimental design should be taken with caution in geriatric clinical practice. Indeed, we sometimes find that carrying out a single trial for a very aged patient can be compared to a quasi-maximal physical effort.

## Conclusion

When walking at comfortable self-selected pace, the standard spatio-temporal walk parameters provided by both the GAITRite® PPC and the GAITRite® CIRFACE seem similar and very highly correlated in older adults with various cognitive or motor status. As a result, it seems possible to us that, for the walk at comfortable self-selected pace condition : (i) the data of studies using these systems can be compared and mixed with a very low risk of bias in a meta-analytic process and, (ii) the geriatric care units can choose the most ergonomic system according to their infrastructure without affecting their gait data. Further studies are needed to confirm our results for other walking conditions.

## Electronic supplementary material

Below is the link to the electronic supplementary material.

Additional Table 1: Comparison between the roll-up and the plate walkways.

## Data Availability

Patient level data are freely available from the corresponding author at DRCI@chu-angers.fr. There is no personal identification risk within this anonymized raw data, which is available after notification and authorization of the competent authorities.

## List of Abbreviations

AD: Alzheimer Disease
CHI: Cognitive Healthy Individual
cm: centimeter
CNIL: National Commission for Information Technology and civil Liberties (CNIL in french)
FAB: Frontal Assessment BatteryHz Hertz
ICC: Intraclass Correlation Coefficient for absolute agreement
LOA: Limits Of Agreement
m: meter
min: minute
Mini GDS: Mini Geriatric Depression Scale
MMSE: Mini Mental State Examination
NCD: NeuroCognitive Disorder
NCT number: ClinicalTrials.gov identifier
PE: Percentage Error
PPS: Platinum Plus Classic
s: second
SD: Standard Deviation

## References

[1] Menz HB, Latt MD, Tiedemann A, Mun San Kwan M, Lord SR (2004). Reliability of the GAITRite® walkway system for the quantification of temporo-spatial parameters of gait in young and older people. Gait Posture.

[2] Park YS, Kim J-W, Kwon Y, Kwon M-S (2018). Effect of age and sex on gait characteristics in the korean Elderly People. Iran J Public Health.

[3] Allali G, Kressig RW, Assal F, Herrmann FR, Dubost V, Beauchet O (2007). Changes in gait while backward counting in demented older adults with frontal lobe dysfunction. Gait Posture.

[4] Allali G, Launay CP, Blumen HM, Callisaya ML, De Cock A-M, Kressig RW (2017). Falls, cognitive impairment, and Gait Performance: results from the GOOD Initiative. J Am Med Dir Assoc.

[5] Godinho C, Domingos J, Cunha G, Santos AT, Fernandes RM, Abreu D (2016). A systematic review of the characteristics and validity of monitoring technologies to assess Parkinson’s disease. J Neuroeng Rehabil.

[6] Rennie L, Löfgren N, Moe-Nilssen R, Opheim A, Dietrichs E, Franzén E (2018). The reliability of gait variability measures for individuals with Parkinson’s disease and healthy older adults – the effect of gait speed. Gait Posture.

[7] Callisaya ML, Blizzard L, Schmidt MD, McGinley JL, Srikanth VK (2008). Sex modifies the Relationship between Age and Gait: a Population-Based study of older adults. The Journals of Gerontology: Series A.

[8] Hollman JH, McDade EM, Petersen RC (2011). Normative spatiotemporal gait parameters in older adults. Gait Posture.

[9] Hollman JH, Youdas JW, Lanzino DJ (2011). Gender differences in dual Task Gait performance in older adults. Am J Mens Health.

[10] Beauchet O, Launay CP, Fantino B, Annweiler C, Allali G. Episodic memory and executive function impairments in non-demented older adults: which are the respective and combined effects on gait performances? AGE 2015;37:70. 10.1007/s11357-015-9812-y. 10.1007/s11357-015-9812-y PMC4497999 26160251

[11] Guedes RC, Dias RC, Pereira LSM, Silva SLA, Lustosa LP, Dias JMD (2014). Influence of dual task and frailty on gait parameters of older community-dwelling individuals. Braz J Phys Ther.

[12] Junior RCF, Porto JM, Rodrigues NC, Brunelli R, de Braga M, de Abreu LFP (2016). Spatial and temporal gait characteristics in pre-frail community-dwelling older adults. Geriatr Gerontol Int.

[13] Roman de Mettelinge T, Cambier D (2015). Understanding the relationship between walking Aids and Falls in older adults: a prospective cohort study. J Geriatr Phys Ther.

[14] Liu H (Howe), McGee M, Wang W, Persson M, editors. Comparison of gait characteristics between older rolling walker users and older potential walker users. Archives of Gerontology and Geriatrics 2009;48:276–80. 10.1016/j.archger.2008.02.004. 10.1016/j.archger.2008.02.004 18359111

[15] Schwenk M, Schmidt M, Pfisterer M, Oster P, Hauer K (2011). Rollator use adversely impacts on assessment of gait and mobility during geriatric rehabilitation. J Rehabil Med.

[16] McGough EL, Logsdon RG, Kelly VE, Teri L (2013). Functional mobility limitations and falls in assisted living residents with dementia: physical performance assessment and quantitative gait analysis. J Geriatr Phys Ther.

[17] Cock A-MD, Fransen E, Perkisas S, Verhoeven V, Beauchet O, Remmen R (2017). Gait characteristics under different walking conditions: Association with the presence of cognitive impairment in community-dwelling older people. PLoS ONE.

[18] McDonough AL, Batavia M, Chen FC, Kwon S, Ziai J (2001). The validity and reliability of the GAITRite system’s measurements: a preliminary evaluation. Arch Phys Med Rehabil.

[19] Webster KE, Wittwer JE, Feller JA (2005). Validity of the GAITRite® walkway system for the measurement of averaged and individual step parameters of gait. Gait Posture.

[20] Werner C, Chalvatzaki G, Papageorgiou XS, Tzafestas CS, Bauer JM, Hauer K (2019). Assessing the concurrent validity of a gait analysis system integrated into a smart walker in older adults with gait impairments. Clin Rehabil.

[21] Domínguez AG, Hochsprung A, Duarte SP, Camino CP, Rodríguez AA, Durán C et al. Study for the Validation of the FeetMe® Integrated Sensor Insole System Compared to GAITRite® System to Assess the Characteristics of the Gait in Patients with Multiple Sclerosis (4038). Neurology 2020;94.

[22] Bethoux F, Varsanik JS, Chevalier TW, Halpern EF, Stough D, Kimmel ZM (2018). Walking speed measurement with an ambient measurement system (AMS) in patients with multiple sclerosis and walking impairment. Gait Posture.

[23] Steinert A, Sattler I, Otte K, Röhling H, Mansow-Model S, Müller-Werdan U (2019). Using New Camera-Based Technologies for Gait Analysis in older adults in comparison to the established GAITRite System. Sens (Basel).

[24] Wang F, Stone E, Skubic M, Keller JM, Abbott C, Rantz M (2013). Toward a passive low-cost in-home gait assessment system for older adults. IEEE J Biomed Health Inform.

[25] Thibaudier Y, Tan AQ, Peters DM, Trumbower RD (2020). Differential deficits in spatial and temporal interlimb coordination during walking in persons with incomplete spinal cord injury. Gait Posture.

[26] Beltramo C, Potentier S, Pourcelle Ph, Dartinet V, Jego A, Kadri N (2005). Profil neuropsychologique des patients âgés hospitalisés: etude prospective dans des unités de court et moyen séjour gériatriques. NPG Neurologie - Psychiatrie - Gériatrie.

[27] de Oliveira Silva F, Ferreira JV, Plácido J, Chagas D, Praxedes J, Guimarães C (2020). Gait analysis with videogrammetry can differentiate healthy elderly, mild cognitive impairment, and Alzheimer’s disease: a cross-sectional study. Exp Gerontol.

[28] Callisaya ML, Launay CP, Srikanth VK, Verghese J, Allali G, Beauchet O (2017). Cognitive status, fast walking speed and walking speed reserve-the Gait and Alzheimer interactions tracking (GAIT) study. Geroscience.

[29] Camicioli R, Licis L (2004). Motor impairment predicts falls in specialized Alzheimer care units. Alzheimer Dis Assoc Disord.

[30] Savica R, Wennberg AMV, Hagen C, Edwards K, Roberts RO, Hollman JH (2017). Comparison of Gait Parameters for Predicting Cognitive decline: the Mayo Clinic Study of Aging. J Alzheimers Dis.

[31] Jayakaran P, DeSouza L, Cossar J, Gilhooly K (2014). Influence of a walking aid on temporal and spatial parameters of gait in healthy adults. PM R.

[32] Folstein MF, Folstein SE, McHugh PR (1975). “Mini-mental state”: a practical method for grading the cognitive state of patients for the clinician. J Psychiatr Res.

[33] Dubois B, Slachevsky A, Litvan I, Pillon B (2000). The FAB: a frontal assessment battery at bedside. Neurology.

[34] D’Ath P, Katona P, Mullan E, Evans S, Katona C (1994). Screening, detection and management of depression in elderly primary care attenders. I: the acceptability and performance of the 15 item geriatric depression scale (GDS15) and the development of short versions. Fam Pract.

[35] Fien S, Henwood T, Climstein M, Rathbone E, Keogh JWL (2019). Gait speed characteristics and their spatiotemporal determinants in nursing home residents: a cross-sectional study. J Geriatr Phys Ther.

[36] Bland JM, Altman DG (1986). Statistical methods for assessing agreement between two methods of clinical measurement. Lancet.

[37] Critchley LA, Critchley JA (1999). A meta-analysis of studies using bias and precision statistics to compare cardiac output measurement techniques. J Clin Monit Comput.

[38] Shrout PE, Fleiss JL (1979). Intraclass correlations: uses in assessing rater reliability. Psychol Bull.

[39] Beauchet O, Allali G, Launay C, Herrmann FR, Annweiler C (2013). Gait variability at fast-pace walking speed: a biomarker of mild cognitive impairment?. J Nutr Health Aging.

[40] Goyal N, Luna G, Curuk E, Aruin AS (2019). Role of motor and cognitive tasks in gait of individuals with mild cognitive impairment. Int J Rehabil Res.

