# Supplementary material for: Comparison of spatio-temporal gait parameters between the GAITRite® platinum plus classic and the GAITRite® CIRFACE among older adults: a retrospective observational study
Source: BMC Geriatr. 2023 Mar 7;23:132. doi: 10.1186/s12877-023-03811-7 (PMC9993600; doi:10.1186/s12877-023-03811-7)
Supplement: Supplementary file 1 — Additional Table 1: Comparison between the roll-up and the plate walkways. [file 12877_2023_3811_MOESM1_ESM.docx]

**Additionnal Table 1: Comparison between the roll-up and the plate walkways.**

**a. Cognitive Healthy Individual**

|  | Roll-up system  GAITRite® PPC  Mean (SD) | Plates system  GAITRite® CIRFACE  Mean (SD) | Bias*  Mean (SD) | 95% LOA | PE |
| --- | --- | --- | --- | --- | --- |
| Velocity (cm/s) | 89.2 ± 23.37 | 90.01 ± 24.12 | 0.81 ± 2.61 | -4.31; 5.92 | 5.7 |
| Cadence (step/min) | 98.65 ± 12.72 | 98.91 ± 13.06 | 0.20 ± 1.08 | -1.91; 2.32 | 2.2 |
| Step time (s) | 0.62 ± 0.11 | 0.62 ± 0.11 | 0.00 ± 0.01 | -0.01; 0.01 | 1.8 |
| Step length (cm) | 53.87 ± 10.52 | 55.01 ± 10.23 | 1.22 ± 1.00 | -0.74; 3.19 | 3.6 |
| Stride time (s) | 1.24 ± 0.21 | 1.23 ± 0.21 | 0.00 ± 0.01 | -0.02; 0.02 | 1.7 |
| Stride length (cm) | 108.21 ± 21.16 | 107.95 ± 21.36 | -0.26 ± 0.79 | -1.81; 1.28 | 1.4 |
| Support base (cm) | 10.54 ± 3.55 | 10.73 ± 3.35 | 0.19 ± 0.78 | -1.34; 1.72 | 14.4 |
| Swing time (s) | 0.42 ± 0.05 | 0.41 ± 0.05 | -0.01 ± 0.01 | -0.03; 0.00 | 8.1 |
| Stance time (s) | 0.81 ± 0.18 | 0.82 ± 0.17 | 0.01 ± 0.01 | -0.01; 0.03 | 5.4 |
| Stride velocity (cm/s) | 90.25 ± 24.01 | 90.35 ± 24.89 | -0.05 ± 1.41 | -4.26; 4.46 | 4.8 |

n = 33; no aid = 29; walking aid = 4; non faller = 7; faller = 26

**b. Minor NeuroCognitive Disorder**

|  | Roll-up system  GAITRite® PPC  Mean (SD) | Plates system  GAITRite® CIRFACE  Mean (SD) | Bias*  Mean (SD) | 95% LOA | PE |
| --- | --- | --- | --- | --- | --- |
| Velocity (cm/s) | 76.48 ± 20.70 | 76.53 ± 20.62 | 0.06 ± 0.81 | -1.52; 1.64 | 2.4 |
| Cadence (step/min) | 95.78 ± 13.82 | 95.89 ± 13.82 | 0.11 ± 0.40 | -0.68; 0.90 | 0.8 |
| Step time (s) | 0.64 ± 0.10 | 0.64 ± 0.10 | 0.00 ± 0.00 | -0.01; 0.01 | 0.8 |
| Step length (cm) | 47.59 ± 9.16 | 49.25 ± 8.13 | 1.66 ± 1.55 | -1.37; 4.70 | 6.3 |
| Stride time (s) | 1.28 ± 0.20 | 1.27 ± 0.20 | 0.00 ± 0.00 | -0.01; 0.01 | 0.7 |
| Stride length (cm) | 95.66 ± 18.30 | 95.36 ± 18.14 | -0.30 ± 0.51 | -1.30; 0.69 | 1.0 |
| Support base (cm) | 11.57 ± 4.38 | 11.54 ± 4.35 | 0.03 ± 0.37 | -0.75; 0.70 | 6.3 |
| Swing time (s) | 0.42 ± 0.06 | 0.41 ± 0.06 | -0.01 ± 0.02 | -0.05; 0.03 | 18.1 |
| Stance time (s) | 0.85 ± 0.16 | 0.86 ± 0.15 | 0.01 ± 0.02 | -0.02; 0.04 | 7.3 |
| Stride velocity (cm/s) | 77.29 ± 20.89 | 77.09 ± 20.79 | -0.20 ± 0.26 | -0.71; 0.31 | 0.7 |

n = 26; no aid = 23; walking aid = 3; non faller = 17; faller = 9

**c. Alzheimer Disease**

|  | Roll-up system  GAITRite® PPC  Mean (SD) | Plates system  GAITRite® CIRFACE  Mean (SD) | Bias*  Mean (SD) | 95% LOA | PE |
| --- | --- | --- | --- | --- | --- |
| Velocity (cm/s) | 76.95 ± 18.39 | 77.59 ± 18.43 | 0.64 ± 1.99 | -3.25; 4.54 | 5.0 |
| Cadence (step/min) | 98.39 ± 12.50 | 98.61 ± 12.49 | 0.22 ± 1.20 | -2.14; 2.57 | 2.4 |
| Step time (s) | 0.62 ± 0.08 | 0.62 ± 0.08 | 0.00 ± 0.01 | -0.02; 0.01 | 2.5 |
| Step length (cm) | 46.67 ± 7.87 | 47.96 ± 7.43 | 1.29 ± 1.04 | -0.75; 3.33 | 4.3 |
| Stride time (s) | 1.23 ± 0.16 | 1.23 ± 0.16 | 0.00 ± 0.01 | -0.03; 0.02 | 2.0 |
| Stride length (cm) | 93.80 ± 15.66 | 93.54 ± 15.46 | -0.27 ± 0.51 | -1.26; 0.73 | 1.1 |
| Support base (cm) | 10.25 ± 3.57 | 10.2 ± 3.51 | -0.04 ± 0.37 | -0.78; 0.69 | 7.2 |
| Swing time (s) | 0.42 ± 0.04 | 0.41 ± 0.05 | -0.01 ± 0.01 | -0.03; 0.01 | 8.6 |
| Stance time (s) | 0.82 ± 0.12 | 0.82 ± 0.12 | 0.01 ± 0.01 | -0.02; 0.03 | 6.2 |
| Stride velocity (cm/s) | 77.86 ± 18.54 | 77.78 ± 18.42 | -0.08 ± 0.83 | -1.71; 1.54 | 2.1 |

n = 36; no aid = 29; walking aid = 7; non faller = 12; faller = 24

**d. No Aid**

|  | Roll-up system  GAITRite® PPC  Mean (SD) | Plates system  GAITRite® CIRFACE  Mean (SD) | Bias*  Mean (SD) | 95% LOA | PE |
| --- | --- | --- | --- | --- | --- |
| Velocity (cm/s) | 84.52 ± 20.80 | 85.20 ± 21.06 | 0.68 ± 2.15 | -3.53; 4.89 | 5.0 |
| Cadence (step/min) | 98.73 ± 12.39 | 98.99 ± 12.56 | 0.27 ± 1.14 | -1.97; 2.50 | 2.3 |
| Step time (s) | 0.62 ± 0.08 | 0.62 ± 0.08 | 0.00 ± 0.01 | -0.01; 0.01 | 2.0 |
| Step length (cm) | 51.1 ± 9.32 | 52.38 ± 8.88 | 1.28 ± 1.11 | -0.90; 3.46 | 4.2 |
| Stride time (s) | 1.23 ± 0.26 | 1.23 ± 0.16 | 0.00 ± 0.01 | -0.02; 0.02 | 1.7 |
| Stride length (cm) | 102.67 ± 18.67 | 102.39 ± 18.66 | -0.28 ± 0.65 | -1.56; 1.0 | 1.3 |
| Support base (cm) | 10.64 ± 3.67 | 10.70 ± 3.57 | 0.07 ± 0.56 | -1.02; 1.15 | 10.2 |
| Swing time (s) | 0.42 ± 0.05 | 0.41 ± 0.05 | -0.01 ± 0.01 | -0.04; 0.01 | 12.0 |
| Stance time (s) | 0.81 ± 0.13 | 0.82 ± 0.12 | 0.01 ± 0.01 | -0.02; 0.04 | 6.42 |
| Stride velocity (cm/s) | 85.52 ± 21.15 | 85.50 ± 21.50 | -0.02 ± 1.52 | -2.99; 2.85 | 3.5 |

n = 81

**e. Walking Aid**

|  | Roll-up system  GAITRite® PPC  Mean (SD) | Plates system  GAITRite® CIRFACE  Mean (SD) | Bias*  Mean (SD) | 95% LOA | PE |
| --- | --- | --- | --- | --- | --- |
| Velocity (cm/s) | 61.15 ± 13.11 | 60.88 ± 12.92 | -0.27 ± 0.28 | -0.81; 0.27 | 0.9 |
| Cadence (step/min) | 92.21 ± 14.62 | 92.06 ± 14.30 | -0.15 ± 0.48 | -1.08; 0.78 | 1.0 |
| Step time (s) | 0.67 ± 0.14 | 0.67 ± 0.14 | 0.00 ± 0.00 | -0.01; 0.01 | 1.1 |
| Step length (cm) | 39.72 ± 5.01 | 41.60 ± 3.87 | 1.88 ± 1.52 | -1.10; 4.86 | 7.3 |
| Stride time (s) | 1.34 ± 0.29 | 1.34 ± 0.28 | 0.00 ± 0.01 | -0.01; 0.01 | 0.8 |
| Stride length (cm) | 79.92 ± 9.94 | 79.98 ± 9.80 | -0.24 ± 0.29 | -0.80; 0.32 | 0.7 |
| Support base (cm) | 11.16 ± 4.61 | 11.06 ± 4.56 | -0.10 ± 0.54 | -1.16; 0.97 | 9.6 |
| Swing time (s) | 0.41 ± 0.06 | 0.40 ± 0.06 | -0.01 ± 0.01 | -0.03; 0.01 | 9.9 |
| Stance time (s) | 0.93 ± 0.24 | 0.94 ± 0.23 | 0.01 ± 0.01 | -0.02; 0.03 | 5.5 |
| Stride velocity (cm/s) | 61.69 ± 13.22 | 61.46 ± 13.02 | -0.22 ± 0.29 | -0.79; 0.35 | 0.9 |

n = 14

**f. Non Faller**

|  | Roll-up system  GAITRite® PPC  Mean (SD) | Plates system  GAITRite® CIRFACE  Mean (SD) | Bias*  Mean (SD) | 95% LOA | PE |
| --- | --- | --- | --- | --- | --- |
| Velocity (cm/s) | 82.18 ± 21.7 | 83.00 ± 21.78 | 0.82 ± 2.12 | -3.33; 4.97 | 5.0 |
| Cadence (step/min) | 96.61 ± 13.44 | 96.96 ± 13.41 | 0.34 ± 1.13 | -1.88; 2.57 | 2.3 |
| Step time (s) | 0.63 ± 0.09 | 0.63 ± 0.09 | 0.00 ± 0.01 | -0.02; 0.01 | 2.3 |
| Step length (cm) | 50.72 ± 8.93 | 52.08 ± 8.52 | 1.36 ± 1.17 | -0.93; 3.66 | 4.5 |
| Stride time (s) | 1.26 ± 0.18 | 1.26 ± 0.18 | 0.00 ± 0.01 | -0.03; 0.02 | 1.9 |
| Stride length (cm) | 101.90 ± 17.83 | 101.63 ± 17.86 | -0.27 ± 0.59 | -1.42; 0.88 | 1.1 |
| Support base (cm) | 10.73 ± 4.13 | 10.87 ± 3.99 | 0.14 ± 0.61 | -1.05; 1.13 | 11.0 |
| Swing time (s) | 0.43 ± 0.05 | 0.42 ± 0.05 | -0.01 ± 0.01 | -0.03; 0.00 | 7.5 |
| Stance time (s) | 0.63 ± 0.14 | 0.84 ± 0.14 | 0.01 ± 0.01 | -0.01; 0.04 | 5.9 |
| Stride velocity (cm/s) | 83.3 ± 22.15 | 83.25 ± 22.15 | -0.05 ± 0.85 | -1.71; 1.61 | 2.0 |

n = 36

**g. Faller**

|  | Roll-up system  GAITRite® PPC  Mean (SD) | Plates system  GAITRite® CIRFACE  Mean (SD) | Bias*  Mean (SD) | 95% LOA | PE |
| --- | --- | --- | --- | --- | --- |
| Velocity (cm/s) | 80.40 ± 21.46 | 80.77 ± 21.97 | 0.37 ± 1.95 | -3.45; 4.19 | 4.7 |
| Cadence (step/min) | 98.47 ± 12.57 | 98.59 ± 12.79 | 0.12 ± 1.04 | -1.93; 2.16 | 2.1 |
| Step time (s) | 0.62 ± 0.10 | 0.62 ± 0.10 | 0.00 ± 0.00 | -0.01; 0.01 | 1.5 |
| Step length (cm) | 48.63 ± 10.12 | 50.01 ± 9.52 | 1.37 ± 1.21 | -1.00; 3.75 | 4.8 |
| Stride time (s) | 1.24 ± 0.19 | 1.24 ± 0.19 | 0.00 ± 0.01 | -0.02; 0.02 | 1.4 |
| Stride length (cm) | 97.74 ± 20.27 | 97.48 ± 20.22 | -0.28 ± 0.63 | -1.52; 0.96 | 1.3 |
| Support base (cm) | 10.71 ± 3.62 | 10.69 ± 3.56 | -0.02 ± 0.52 | -1.02; 0.99 | 9.5 |
| Swing time (s) | 0.41 ± 0.04 | 0.40 ± 0.05 | -0.01 ± 0.01 | -0.04; 0.02 | 13.7 |
| Stance time (s) | 0.82 ± 0.16 | 0.83 ± 0.15 | 0.01 ± 0.01 | -0.02; 0.04 | 6.5 |
| Stride velocity (cm/s) | 81.22 ± 21.80 | 81.17 ± 22.28 | -0.05 ± 1.66 | -3.21; 3.21 | 4.0 |
| n = 59  * Difference between Roll-up and Plates system (Plates-Roll-up)  LOA : Limits Of Agreement  PE : Percentage Error  cm: centimeter; m: meter; min: minute; s: second  ICC : Intraclass Correlation Coefficient for absolute agreement  All Pearson correlations and ICCs were significant at P <.001. | | | | | |
